# Nitric Acid-Treated Carbon Fibers with Enhanced Hydrophilicity for *Candida tropicalis* Immobilization in Xylitol Fermentation

**DOI:** 10.3390/ma9030206

**Published:** 2016-03-17

**Authors:** Le Wang, Na Liu, Zheng Guo, Dapeng Wu, Weiwei Chen, Zheng Chang, Qipeng Yuan, Ming Hui, Jinshui Wang

**Affiliations:** 1 School of Biological Engineering, Henan University of Technology, Zhengzhou 450001, China; wanglely1984@163.com (L.W.); liuna1981@126.com (N.L.); chenweiw4054@foxmail.com (W.C.); huiming69@126.com (M.H.); 2 College of Textile, Zhongyuan University of Technology, Zhengzhou 450001, China; huiyi1981@163.com; 3 School of Chemistry and Chemical Engineering, Henan Normal University, Xinxiang 453001, China; 4 State Key Laboratory of Chemical Resource Engineering, Beijing University of Chemical Technology, Beijing 100029, China; changzheng@mail.buct.edu.cn

**Keywords:** carbon fiber (CF), oxidation treatment, surface properties, cell immobilization, xylitol fermentation

## Abstract

Nitric acid (HNO_3_)-treated carbon fiber (CF) rich in hydrophilic groups was applied as a cell-immobilized carrier for xylitol fermentation. Using scanning electron microscopy, we characterized the morphology of the HNO_3_-treated CF. Additionally, we evaluated the immobilized efficiency (IE) of *Candida tropicalis* and xylitol fermentation yield by investigating the surface properties of nitric acid treated CF, specifically, the acidic group content, zero charge point, degree of moisture and contact angle. We found that adhesion is the major mechanism for cell immobilization and that it is greatly affected by the hydrophilic–hydrophilic surface properties. In our experiments, we found 3 hto be the optimal time for treating CF with nitric acid, resulting in an improved IE of *Candida tropicalis* of 0.98 g∙g^−1^ and the highest xylitol yield and volumetric productivity (70.13% and 1.22 g∙L^−1^∙h^−1^, respectively). The HNO_3_-treated CF represents a promising method for preparing biocompatible biocarriers for multi-batch fermentation.

## 1. Introduction

Utilization of immobilized cells eliminates time-consuming and cost-intensive steps involved in the isolation and purification of intracellular enzymes [1]. Immobilization with biocompatible carriers prevents microbial cells from being diluted in an open-water system and extends the production capacity of microbial cells [2]. The carrier material provides a protective surface and pore spaces for bioremediating microbial cells, creating favorable conditions for the batches’ fermentation [3]. Currently, research is investigating the maintenance of high and long-lasting cell activity using different cell immobilization carriers for efficient and continuous fermentation operations [4,5].

The selection of the immobilization carrier is crucial in cell immobilization. The surface properties of the carriers greatly affected the growth and metabolism of immobilized cells [6]. Ideally, the carrier material should be biodegradable, available in large quantities at low cost, and also exhibit appropriate physical properties for the sufficient adherence of specific microorganisms [7]. Previously, different materials such as porous glass, Ca-alginate, chitosan and sodium alginate have been investigated as immobilized cell carriers for microorganisms in xylitol fermentation. Carbon has been widely used in medicine, bioengineering and biomaterials, owing to its good biocompatibility [8,9,10,11]. The biocompatibility of carbon fiber (CF) was shown to possess a high capacity for bacterial adhesion and has been adopted as a biofilm support for wastewater treatment [12,13,14,15]. Therefore, carbon-based materials have great potential for application as immobilization carriers for multi-batch fermentation.

The major forms of cell immobilization could be classified asfour categories: adhesion, entrapment, containment, and aggregation [16]. Cell adhesion is closely related to the surface properties (wettability, polarity, surface energy, electrical properties, morphology and roughness, and chemical composition) of the materials and requires further optimization by different surface treatments [17,18].

Some reports show that the adhesion capacity of cells of microorganisms could be improved by a material surface with moderate hydrophilicity [19,20,21,22]. CF usually possess a nonpolar surface due to the high-temperature conditions involved in manufacturing it, which is not beneficial to its utilization as the carrier. Zhu *et al.* [23] show that the hydrophilicity and biocompatibility of carbon materials is enhanced by increasing the content of surface oxygenic functional groups. The oxygen-based functional groups of carbon-based materials (such as carbon fiber, activated carbon, carbon nanotube, *etc.*) were increased by the surface oxidation treatment [12,24,25,26]. Bao and Dai [14] also demonstrate that theinorganic acid treatment is an efficient way to enhance the hydrophilicity of the carbon-based materials.

Nitric acid (HNO_3_)-treated CF is seldom used as immobilized carrier in multi-batch xylitol fermentation. Here, the surface morphology, the content of acidic groups, point of zero charge (pH_pzc_), degree of moisture (DM), and contact angle (CA) of the HNO_3_-treated CF was evaluated to study the effects on immobilized efficiency (IE), xylitol yield and volumetric productivity in thebatchesfermentation of xylitol by *Candida tropicalis* (*C. tropicalis*).

## 2. Results and Discussion

### 2.1. SEM of Raw and Treated CF

**Figure 1:**
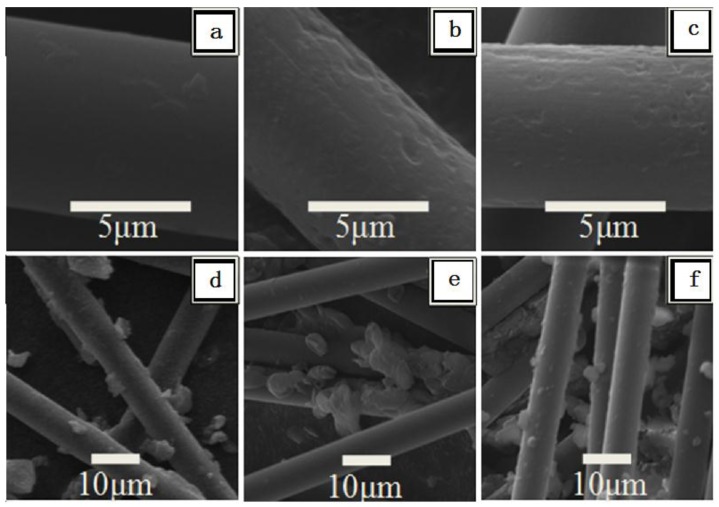
Scanning electron microscopy (SEM) images of carbon fibercarrier with raw and treated morphology and immobilized *C. tropicalis* (**a**) CF carrier without oxidation by nitric acid; (**b**) CF carrier after exposure to nitric acid treatment for 3 h; (**c**) CF carrier after exposure to nitric acid treatment for 6 h; (**d**) CF carrier without treatment immobilized with *C. tropicalis*; (**e**) Immobilized *C. tropicalis* by CF carrier treated with nitric acid for 3 h; (**f**) Immobilized *C. tropicalis* by CF carrier treated with nitric acid for 6 h.

Figure 1 shows the scanning electron microscopy (SEM) images of the CF carrier with and without nitric acid treatment. The surface of Raw-CF is reasonably smooth and regular (Figure 1a). Increasing exposure time to nitric acid created grooves and black etch pits (Figure 1b,c). The grooves on the CF carrier were formed by removing the amorphous carbon during the nitric acid treatments, which may help to increase the roughness and binding sites on material surface [18]. Figure 1 also shows images of cells immobilized by CF with and without nitric acid treatment, after three batches of xylitol production. Only a few cells were immobilized onto the surface of Raw-CF and CF-N_h6_. When comparing cell immobilization with raw (Figure 1d) and treated CF (Figure 1e,f), more cells were immobilized on the treated CF surfaces. This phenomenon was consistent with previous reports that adhesion is the main mechanism by which cells are immobilized on a carrier [15,16,23]. Yet, despite the similar surface morphology of CF-N_h3_ and CF-N_h6_,more cells adhered to CF-N_h3_ than CF-N_h6_ carrier, after the batches’ fermentation immobilized with *C. tropicalis* (Figure 1e,f), which suggests that the capacity of cell immobilization could be improved after the treatment by nitric acid and the amount of treated time has an optimum value.

### 2.2. Effects of Nitric Acid Treatment on Surface Properties of CF and IE

The contents of surface total acidic functional groups on CF are shown in Table 1. With increasing exposure to nitric acid, the surface oxidation introduced more acidic groups on CF, which increased from 0.244 to 1.38 mmol∙g^−1^.The content of acidic functional groups in CF-Nh_3_ was higher than those in Raw-CF. The increase in acidic functional groups was ascribed to the formed action of oxygenated functional groups, with the removal of loosely bonded defective surface layer of carbon and the oxygen chemisorption proceeds on the edge carbon atoms in the oxidative treatment of CF [27].

**Figure 2:**
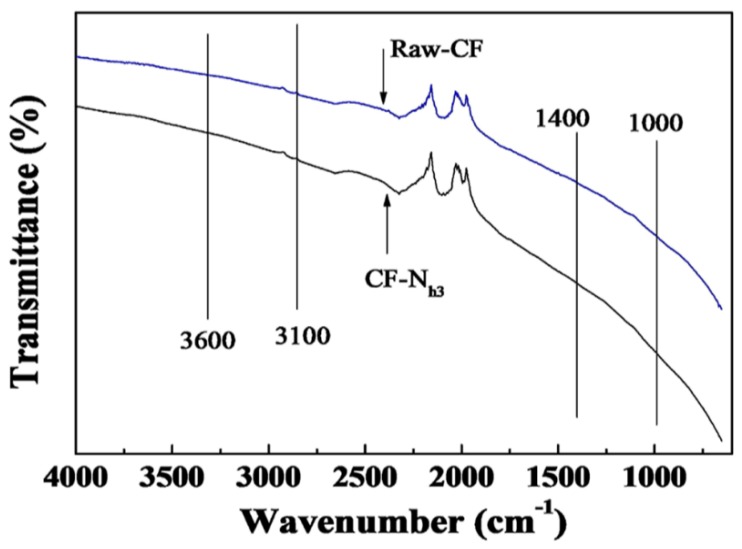
FTIR spectrum of Raw-CF and CF-N_h3_.

The spectra of the Raw-CF and CF-N_h3_ samples were recorded between 4000 and 400 cm^−1^ using a Nicolet 380 FTIR, and were depicted in Figure 2. OH stretching vibrations are reflected by the wide peak between 3600 cm^−1^ and 3100 cm^−1^ [28]. The groups of C-O-C, C-O-N or C-N appeared between 1400 cm^−1^ and 1000 cm^−1^ [8]. The peaks of CF-N_h3_ are stronger than those of Raw-CF, suggesting that oxygen functional groups are successfully introduced through oxidation by nitric acid.

Results represent the average of two independent experiments conducted with three replicates for each condition.

**Figure 3:**
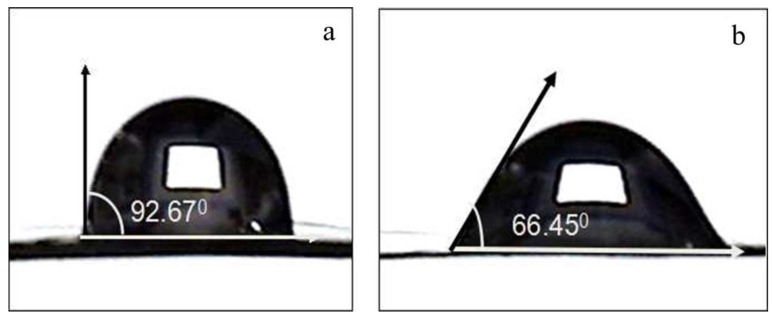
The image of contact angles on Raw-CF and CF-N_h3_ (**a**) Results and image of contact angles on Raw-CF; (**b**) Results and image of contact angles on Raw-CF.

In Table 2, the data of DM, pH_pzc_ and CA were significantly different (*p* < 0.05), respectively. The hydrophilicities of HNO_3_-treated CFs were evaluated by measuring both DM and CA. With an increase of the duration of nitric acid treatment, the DM values increased gradually from 1.68% to 18.7% and the values of CA decreased from 92.67° to 53.49°. This was attributed to the introduction of hydrophilic functional groups. The wettability of CF-N_h3_ increased compared to Raw-CF (in Figure 3). The content of acidic functional groups correlated with the DM relationship, which improves the surface wettability. In the previous literature, it was suggested that the hydrophilicity of materials increased as the number of oxygen functional groups increased [14,23]. Therefore, nitricacid oxidation was a simple and effective way to improve the hydrophilicity of CF.

Additionally, the pH_pzc_ values of CF decreased from 5.93 to 2.71 as treatment time increased (Table 2). We attribute this to the increase in acidic functional group content in CF. Moreover, the microenvironment around the surface of CF is changed by the pH_pzc_, influencing the growth of the cell.

**Figure 4:**
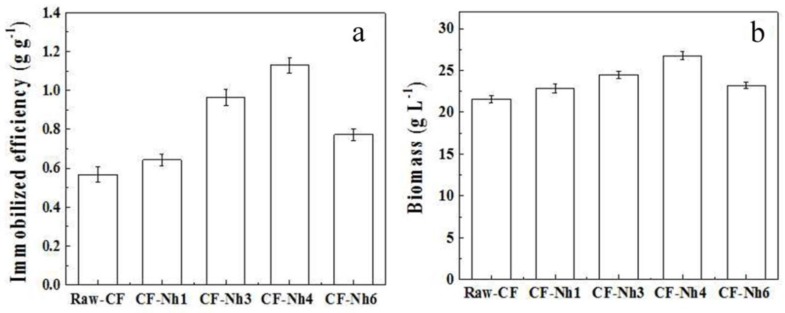
Effects of oxidation carriers on immobilized efficiency (IE) and biomass of fermentation (**a**) Effects of oxidation carriers on the IE; (**b**) Effects of oxidation carriers on the biomass of fermentation. Biomass was calculated using the amount of free cells and immobilized cells in the third fermentation by immobilized C. tropicalis on CF with and without treatment.

As shown in Figure 4a, the IE initially increased from 0.56 g∙g^−1^ to 1.13 g∙g^−1^ when CF is treated for 1–4 h, and decreased to 0.773 g∙g^−1^ when treated for6 h later. As the treatment time increased, a higher IE of the cell was acquired by the treated CF compared with the Raw-CF. The results of biomass concentration in multi-batch fermentation with and without treated CF immobilization were also investigated (Figure 4b). The maximum and minimum biomasses were obtained by fermentation with CF-N_h4_ and Raw-CF, with biomass values of 26.77 g∙L^−1^ and 21.56 g∙L^−1^, respectively. Fermentation with CF-N_h3_ resulted in the biomass of 24.47 g∙L^−1^. Cell immobilization could lead to a higher biomass and enhance biological stability [3], which are conducive to the continuous xylitol fermentation. The observation of increased biomass was potentially because the properties of the immobilization material were changed by oxidation. In addition, the treated CF provided a non-toxic and profitable microenvironment for cell growth.

Figure 4a and Table 1 show that the IE increased at first and then gradually decreased as the hydrophilicity of the carriers was further increased. According to the Derjaguin-Landau-Verwey-Overbeek (DLVO) theory for bacterial adhesion, the total interaction between a surface and a cell is the summation of their Van der Waals and Coulomb interactions [29]. The Van der Waals attractive force is dominant in the vicinity of a surface. Cells cannot separate from the surface by Brownian motion. Thus, the Van der Waals force between the cell and CF surface was enhanced with the moderate surface hydrophilicity [21].In addition, another reason for the hydrophilicity of cells, which was consistent with the findings of a thermodynamic approach that suggests yeast with a hydrophilic cell surface prefer adhering to the surfaces of hydrophilic materials. In contrast, the Coulomb interaction becomes dominant when electrostatic repulsion (repulsiveforce of the Coulomb interaction) increases owing to the presence of a greater number of negatively charged functional groups on the CF surface. Therefore, the increase in electrostatic repulsion between the CF and cell surface resulted in a decrease in IE. We demonstrate here that using adhesion as the major mechanism of the immobilization of *C. tropicalis* can be controlled by the hydrophilic–hydrophilic surface properties and the electrostatic interactions. This conclusion is consistent with previous reports that the overall cell wall charge is not the principal determinant in cell adhesion [30]. In addition, oxidation with nitric acid for 3–4 h is the optimum treatment time to improve the properties of CF as a carrier to immobilized *C. tropicalis*.

Results of DM and pH_pzc_ represent the average of two independent experiments conducted with three replicates for each condition, and the values of CA are based on 5–7 measurements.

Results represent the average of two independent experiments conducted with three replicates for each condition.

### 2.3. Multi-Batch Fermentation of Xylitol by C. tropicalis Immobilized on Treated CF

In the multi-batch xylitol fermentation, the differences of the IE, biomass and xylitol yields were statistically significant (*p* < 0.05), respectively. The maximum xylitol yield of 70.13 g∙L^−1^ and volumetric productivity of 1.22 g∙L^–1^∙h^–1^ were obtained in the second batch of fermentation with CF-N_h3_ immobilization (Table 3). The xylitol yield with CF-N_h3_ fermentation were improved 11.43%, and 8.21%, respectively, comparing with those of Raw-CF and free fermentation. The volumetric productivity with CF-N_h3_ fermentation were improved 0.2 g∙L^–1^∙h^−1^, and 0.14∙g∙L^−1^∙h^−1^, respectively, comparing with those of Raw-CF and free fermentation. In addition, the average xylitol yield and volumetric productivity with CF-N_h3_ fermentation were improved by approximately 8%, compared with that of CF-Nh_4_.

**Figure 5:**
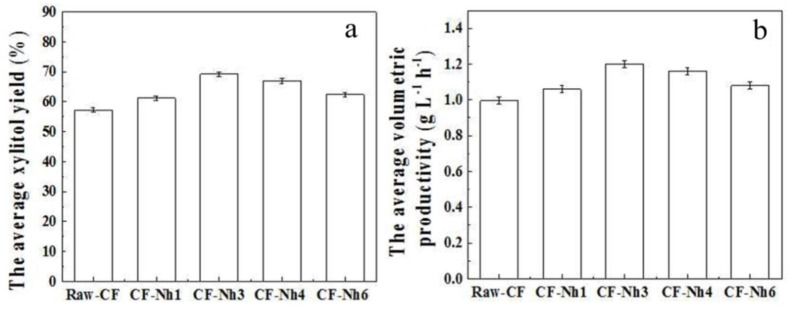
Effect of oxidation time on the multi-batch xylitol fermentation (**a**) Effects of oxidation time on the average xylitol yield of fermentation batches; (**b**) Effect of oxidation time on the average volumetric productivity of the fermentation batches.

When the IE and total biomass increased from 0.568 to 0.98 g∙g^−1^ and 20.6 to 26.5 g∙L^−1^, respectively (Figure 4), the average xylitol yield and the average volumetric productivity of xylitol increased from 57.28 to 69.17 g∙g^−1^ and from 0.998 to 1.20 g∙L^−1^∙h^−1^, respectively (Figure 5). This suggests that xylitol fermentation is closely related to the IE, which could be attributed to an increase in the metabolic activity of the immobilized cells [31,32,33]. In addition, the mildly acidic microenvironment provided by pH_pzc_ stimulates cells to produce more ATP, which increases biomass fermentation [32].

Results of average xylitol yield and average volumetric productivity for three batches of multi-batch xylitol fermentation by *Candida tropicalis* immobilized on the CF with and without treatment.

The CF-N_h4_ obtained the highest values of IE (1.13 g∙g^−1^) during the immobilized fermentation (Figure 4a). Xylitol yield and volumetric productivity reached their highest respective values of 68.86 g∙g^−1^ and 1.16 g∙L^−1^∙h^−1^ during immobilized fermentation with CF-N_h3_ (Figure 5). However, the biomass and IE of CF-N_h4_ were higher by 0.2 g∙L^−1^ and 0.18 g∙g^−1^, respectively, than those of CF-N_h3_ (Figure 4b). We attribute this to the decreased dissolved oxygen in the medium and the increased nutrient supplementation that can maintain the physiological activity of cells immobilized by CF-N_h4_ in fermentation. Hence, the efficiency of internal and external mass transfer decreased during fermentation, resulting in a decrease in nutrient supply capacity [3,34]. Therefore, 3 h is the optimal time for nitric acid treatment to immobilize *C. tropicalis* in the multi-batch xylitol fermentation.

Moreover, total biomass decreased as the value of pH_pzc_ decreased from 4.02 (CF-N_h3_) to 2.71 (CF-N_h6_) (Table 2 and Figure 4b), and the average yield of fermentation with CF-N_h6_ was approximately 10% lower than CF-N_h3_ (Figure 4a and Figure 5a). This may result from the increased H^+^ concentration in the microenvironment as the pH_pzc_ decreases. The plasma membrane ATPase, which pumps protons out of the cell at the expense of ATP hydrolysis, might thus be compensating for the decrease in the intracellular pH. Therefore, less ATP inhibits biomass formation, decreasing xylitol fermentation [32]. The highest xylitol yield and volumetric productivity were obtained in the immobilized fermentation of CF-N_h3_ with the IE of 0.98 g∙g^−1^ and pH_pzc_ values of 4.02. It results from the weak acid broth and the moderate total cell concentration, which benefits the producing of ATP and enhances the transfer efficiency of nutrient [33]. The activity of immobilized cells could be influenced by the microenvironment provided by the carrier [3]. Therefore, the oxidation treatment of CF by nitric acid for 3 h is effective to improve the immobilized fermentation of xylitol.

## 3. Experimental Section

### 3.1. Immobilization Carriers

The length of CF (reinforcement cloth bought from Shanghai Yingjia Special Fiber Material Co., LTD, Shanghai, China) was cut into 0.5 cm pieces and soaked in acetone for 3 h. The CF was then boiled for 3 h in deionized water, which was replaced every 0.5 h [23]. The product (denoted as Raw-CF) was put into a desiccator and dried at 120 °C for 2 h before utilization or additional treatment. The chemical oxidation treatment of the CF surface was performed using 68.3% nitric acid (all reagents were of the highest analytical grade; Tianjin Kermel Chemical Reagent Co., LTD, Tianjin, China) for 1–6 h. Moreover, the products were denoted as CF-N_h1_, CF-N_h2_, CF-N_h3_, CF-N_h4_, CF-N_h5_ and CF-N_h6_. The CF surface was then washed with deionized water until the pH value was about 7.0. The samples were dried at 120 °C for 2 h. Finally, the CFs were mixed with the medium by the dosage of 1% (w∙v^−1^) and sterilized at 120 °C for 20 min in the autoclave.

### 3.2. Multi-Batch Xylitol Fermentation by Immobilized Cells

*Candida tropicalis* was purchased from China General Microbiological Culture Collection Center (Beijing, China). The strain was cultured on an agar slant containing 8 g∙L^−1^ yeast extract, 4 g∙L^−1^ glucose, 10 g∙L^−1^ xylose and 20 g∙L^−1^ agar at 30 °C for 48 h and stored at 4 °C [3]. Cells from the slant were aseptically inoculated to 50 mL of inoculum medium in 250 mL flasks at 30 °C for 24 h on 180 rpm shaker. The inoculum medium includes 20 g∙L^−1^ xylose, 20 g∙L^−1^ glucose, 10 g∙L^−1^ yeast extract, 3 g L^−1^ KH_2_PO_4_ and 2 g L^−1^ (NH_4_)_2_HPO_4_. The prepared inoculum was directly added into the culture medium with a 6% of volume fraction. Culture medium used for xylitol fermentation was 15 g∙L^−1^ glucose, 80 g∙L^−1^ xylose, 8 g∙L^−1^ yeast extract, 0.4 g∙L^−1^ MgSO_4_.7H_2_O, 3 g∙L^−1^ KH_2_PO_4_.

At the beginning of first batch fermentation, the flask containing 50 mL of the culture medium with a certain dosage of CF was incubated at 30 °C on an orbital shaker agitated at 180 rpm for 24 h. The cultivation was continued, adjusting speed at 150 rpm. At the end of the first batch, two-thirds of culture medium was removed from the flasks, leaving the CF carriers with immobilized cells and few free cells for the second batch. After adding the same volume of fresh feed medium to 50 mL, similarly to the culture media, except for 20 g∙L^−1^ glucose, the second batch started under the same conditions as the first batch. The next batch was prepared in the same manner.

### 3.3. Measurements

The surface morphologies of CFs after the treatment of chemical oxidation for 0–6 h and CFs as the carrier of immobilized *C. tropicalis* in the xylitol fermentation were observed by scanning electron microscopy (SEM). The dried specimens were carefully mounted onto aluminum stubs using conductive adhesive. The mounted specimens were sputter-coated with gold and examined using a scanning electron microscope (JSM-6380 LV, JEOL Ltd, Tokyo, Japan).

The surface acidity was estimated by mixing 0.20 g of CF with 25 cm^3^ of 0.05 M NaOH solution in a closed flask and agitating for 48 h at room temperature. The suspension was decanted and the remaining NaOH was titrated with 0.05 M HCl.

The Fourier transform infrared spectroscopes were recorded on a Nicolet 380 Fourier transform infrared spectroscopy (FTIR) (Thermo Fisher Scientific, Waltham, MA, USA) by using pressed KBr pellets.

pH_pzc_ was measured by adjusting the pH of 50 mL 0.01 M NaCl solution to between 2 and 12, then 0.15 g of CF was added and the final pH was measured after 48 hours of agitation at room temperature. pH_pzc_ is the point at which pH_initial_ – pH_final_ = 0.

The surface hydrophilicity of the CF was represented by the DM defined as the weight (g) of water adsorbed per gram CF after the CF surface has hung in a closed container with a saturated solution of ammonium sulfate in the bottom for 24 h (85% relative humidity) [14].

CA measurements were determined at 25 °C by a contact angle meter (YIKE-360A, ChengdeYike Test Instrument Factory, Chengde, China) employing 1 μL drops of pure deionized water. The readings were stabilized and taken 120 s after droppings. The CA of the liquid was measured using a CA analyzer equipped with a high-speed camera [35].

The IE was defined as the biomass (g) immobilized on 1 g of CF carrier in the medium until the end of fermentation. The samples were centrifuged for 15 min at 3000× *g*. Then the supernatants were analyzed for xylitol and xylose. The free cells and immobilized cellswere oven dried at 80 °C for 24 h to a constant weight. The biomass contained the free cells and immobilized cells.

All samples were filtered through 0.22 μm filters and diluted prior to HPLC analysis. The analysis was performed using a Hitachi HPLC system (Hitachi, Tokyo, Japan) and the N2000 software (Ejer Technol. Co. Ltd, Zhejiang, China). Xylitol and xylose were measured on a Sugar-pak1 column (Waters, Milford, MA, USA) at 80 °C, with the mobile phase of ultra-pure water supplied at a flow rate of 0.5 mL∙min^−1^. Xylitol yield (%) is expressed as the ratio between the final xylitol concentration and the initial xylose concentration in the broth.

### 3.4. Statistical Data Treatment

The experiment results were statistically analyzed using the software of SPSS 17.0 (SPSS, Chicago, IL, USA). Data were analyzed using ANOVA and means were considered to be significantly different at *p* < 0.05 as determined by least significant differences (LSD).

## 4. Conclusions

Our research shows that HNO_3_ oxidation is a favorable surface treatment for CF carriers. We found adhesion to be the major mechanism for cell immobilization, which was greatly affected by the surface properties of CF. Based on our controlled experiments, the optimal nitric acid treating time was 3 h, resulting in an improved IE of the cell of 0.98 g∙g^−1^, with the highest xylitol yield and volumetric productivity (70.13% and 1.22 g∙L^−1^∙h^−1^, respectively). The HNO_3_-treated CF represents a promising method to prepare biocompatible material as immobilized biocarriers in the industrialization of continuous xylitol production.

## Figures and Tables

**Table 1:** The content of total acidic functional groups in raw and treated carbon fibers.

Carriers Properties	Raw-CF	CF-N_h1_	CF-N_h3_	CF-N_h6_
Total acidity (mmol∙g^−1^)	0.244 ± 0.011	0.365 ± 0.014	0.949 ± 0.023	1.38 ± 0.015

**Table 2:** The properties in raw and treated CF carriers.

Carriers Properties	Raw-CF	CF-N_h1_	CF-N_h3_	CF-N_h4_	CF-N_h6_
DM (%)	1.68 ± 0.21	2.77 ± 0.25	9.66 ± 0.16	12.8 ± 0.20	18.7 ± 0.22
CA (°)	92.67 ± 1.14	77.02 ± 1.53	66.45 ± 1.86	59.31 ± 1.43	53.49 ± 1.61
pH_pzc_	5.93 ± 0.03	5.45 ± 0.01	4.02 ± 0.01	3.88 ± 0.01	2.71 ± 0.04

**Table 3:** Yield and volumetric productivity in the multi-batch fermentation of xylitol.

Carrier Fermentation	Free	Raw-CF	CF-N_h1_	CF-N_h3_	CF-N_h4_	CF-N_h6_
YPS^−1^ (%) ^1^	57.76± 0.1	57.6 ± 0.1	60.11 ± 0.1	68.66 ± 0.1	67.51 ± 0.1	60.74 ± 0.1
QP (g∙L^−1^∙h^−1^) ^2^	1.00 ± 0.01	1.00 ± 0.01	1.05 ± 0.02	1.19 ± 0.01	1.17 ± 0.01	1.06 ± 0.01
YPS^−1^ (%) ^3^	61.92 ± 0.1	58.7 ± 0.1	61.77 ± 0.1	70.13 ± 0.1	68.7 ± 0.1	64.19 ± 0.2
QP (g∙L^−1^∙h^−1^) ^4^	1.08 ± 0.02	1.02 ± 0.01	1.07 ± 0.01	1.22 ± 0.02	1.19 ± 0.01	1.12 ± 0.01
YPS^−1^ (%) ^5^	60.87 ± 0.1	55.5 ± 0.1	61.54 ± 0.1	68.73 ± 0.1	64.38 ± 0.1	62.11 ± 0.1
QP (g∙L^−1^∙h^−1^) ^6^	1.06 ± 0.01	0.97 ± 0.02	1.07 ± 0.01	1.20 ± 0.01	1.12 ± 0.02	1.08 ± 0.01

^1^ Xylitol yield; and ^2^ volumetric productivity in the first batch of fermentation by *C. tropicalis* immobilized on CF with and without treatment; ^3^ Xylitol yield and ^4^ volumetric productivity in the second batch of fermentation by *C. tropicalis* immobilized on CF with and without treatment; ^5^ Xylitol yield and ^6^ volumetric productivity in the second batch of fermentation by *C. tropicalis* immobilized on CF with and without treatment. Results represent the average of two independent experiments conducted with three replicates for each condition.
